# Song exposure regulates known and novel microRNAs in the zebra finch auditory forebrain

**DOI:** 10.1186/1471-2164-12-277

**Published:** 2011-05-31

**Authors:** Preethi H Gunaratne, Ya-Chi Lin, Ashley L Benham, Jenny Drnevich, Cristian Coarfa, Jayantha B Tennakoon, Chad J Creighton, Jong H Kim, Aleksandar Milosavljevic, Michael Watson, Sam Griffiths-Jones, David F Clayton

**Affiliations:** 1 Department of Biology and Biochemistry, University of Houston, Houston, Texas 77204, USA; 2 Departments of Pathology, Baylor College of Medicine, Houston, Texas 77030, USA; 3 Human Genome Sequencing Center, Baylor College of Medicine, Houston, Texas 77030, USA; 4 Department of Cell and Developmental Biology, University of Illinois, Urbana-Champaign, IL 61801, USA; 5 W.M. Keck Center for Comparative and Functional Genomics, Roy J. Carver Biotechnology Center, University of Illinois, Urbana-Champaign, IL 61801, USA; 6 Dan Duncan Cancer Center, Baylor College of Medicine, Houston, TX 77030, USA; 7 ARK-Genomics, The Roslin Institute and R(D)SVS, University of Edinburgh, Easter Bush, EH25 9RG, UK; 8 Faculty of Life Sciences, University of Manchester, Manchester, M13 9PT, UK; 9 Institute for Genomic Biology, University of Illinois, Urbana-Champaign, IL 61801, USA; 10 Beckman Institute, University of Illinois, Urbana-Champaign, IL 61801, USA; 11 Bioinformatics Research Laboratory (BRL), Department of Molecular & Human Genetics, Baylor College of Medicine, Houston, TX 77030, USA

## Abstract

**Background:**

In an important model for neuroscience, songbirds learn to discriminate songs they hear during tape-recorded playbacks, as demonstrated by song-specific habituation of both behavioral and neurogenomic responses in the auditory forebrain. We hypothesized that microRNAs (miRNAs or miRs) may participate in the changing pattern of gene expression induced by song exposure. To test this, we used massively parallel Illumina sequencing to analyse small RNAs from auditory forebrain of adult zebra finches exposed to tape-recorded birdsong or silence.

**Results:**

In the auditory forebrain, we identified 121 known miRNAs conserved in other vertebrates. We also identified 34 novel miRNAs that do not align to human or chicken genomes. Five conserved miRNAs showed significant and consistent changes in copy number after song exposure across three biological replications of the song-silence comparison, with two increasing (tgu-miR-25, tgu-miR-192) and three decreasing (tgu-miR-92, tgu-miR-124, tgu-miR-129-5p). We also detected a locus on the Z sex chromosome that produces three different novel miRNAs, with supporting evidence from Northern blot and TaqMan qPCR assays for differential expression in males and females and in response to song playbacks. One of these, tgu-miR-2954-3p, is predicted (by TargetScan) to regulate eight song-responsive mRNAs that all have functions in cellular proliferation and neuronal differentiation.

**Conclusions:**

The experience of hearing another bird singing alters the profile of miRNAs in the auditory forebrain of zebra finches. The response involves both known conserved miRNAs and novel miRNAs described so far only in the zebra finch, including a novel sex-linked, song-responsive miRNA. These results indicate that miRNAs are likely to contribute to the unique behavioural biology of learned song communication in songbirds.

## Background

Songbirds are important models for exploring the neural and genomic mechanisms underlying vocal communication, social experience and learning (reviewed in [1]). Songbirds communicate using both innate calls and learned vocalizations (songs), and unique specializations of the brain evolved to support this behavior (reviewed in [2]). In the zebra finch, only the male produces songs, although both sexes process and discriminate specific songs [3-6]. The genome is actively engaged by song communication, as first shown in an early demonstration of how gene responses in the brain discriminate among different song stimuli [7]. The genomic response is not a simple correlate of neural activity and it can vary significantly according to the salience and behavioral context of the experience [8-13]. Recent studies using microarray technology have now shown that song exposure affects the expression of thousands of genes in the auditory forebrain [14,15]. Repeated exposure to one song leads to an altered gene expression profile, correlated with habituation of both the behavioral and immediate genomic responses to that specific song. These observations suggest the involvement of large and dynamic transcriptional network in the recognition and memory of complex vocal signals [14].

MicroRNAs (miRNAs or miRs) are emerging as potential control points in transcriptional networks, and may be particularly important for the evolution of brain and behavior. Many miRNAs are expressed in the brain [16], often in different patterns in different species [17-19]. Brain miRNAs undergo dramatic changes in expression during development [20-22] and aging [23] and have been functionally implicated in neurological disease [24]. They may also function in the normal physiological operation of the nervous system as suggested by evidence for involvement of miR-132 and miR-219 in circadian clock regulation [25] and miR-134 in control of dendritic translation [26,27].

Here we apply massively parallel Illumina sequencing to probe the involvement of miRNAs in the processing of song experience in the zebra finch auditory forebrain. We begin by identifying 155 different miRNA sequences and the genomic loci of their precursor sequences in the zebra finch genome, including 34 miRNA genes that have not been detected in the genomes of other species. We then ask whether the miRNA content changes after song exposure and find robust evidence of miRNA responses to song playbacks. We also assess correlations between expression changes of a novel miRNA and its predicted target mRNAs during song habituation. The results indicate an active role for miRNAs in the neural processing of a natural perceptual experience - hearing the sound of another bird singing.

## Results

### The miRNAs of the zebra finch auditory forebrain

We carried out Illumina small RNA sequencing (RNA-seq) on the small RNA (~18-30 nucleotides) fraction of total RNA isolated from adult zebra finch auditory forebrain. Ultimately, we performed 6 Illumina runs on 6 different RNA samples, to assess the effects of song exposure (next section). First we describe the overall small RNA profile obtained by combining the results of all the runs, representing 36 adult zebra finches (equal numbers of males and females). A total of 20 million reads were obtained (Table 1) and aligned to reference miRNA sequences from other species (miRBase version 13.0). Overall we identified 107 non-redundant miRNAs representing 52% of sequences that have been previously identified in chicken, rodent and human. The remaining sequences mapping to the piRNA database were denoted as piRNA reads (~30%) (Additional File 1, Table S1).

**Table 1:** Summary statistics for the read alignments

		Male silence	Male song	Female silence	Female song	Mix silence	Mix song
		
Total Reads		2,704,778	2,056,391	3,173,108	3,546,038	3,962,050	4,738,528
		
Total Usable Reads		1,179,330	1,155,168	2,244,376	2,498,648	2,249,188	2,950,398
Reads aligning with	Total	401,934	209,944	1,638,528	1,755,748	1,348,109	2,113,006
	
known miRNA	Fraction	34%	18%	73%	70%	60%	72%

Six different pools of auditory forebrain were analyzed independently by Illumina small RNA sequencing, as described in the text.

**Figure 1:**
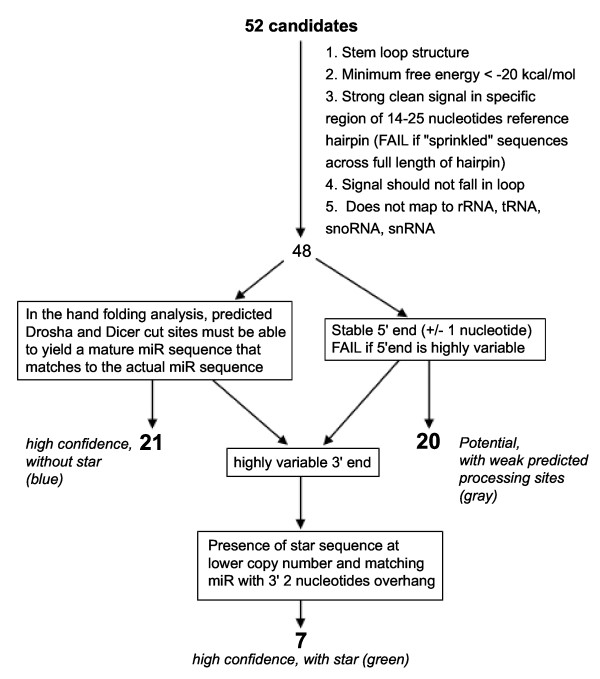
**Pipeline with yields for analysis of putative novel miRNAs**. 52 small RNA sequences did not align to miRBase reference sequences and were assessed for miRNA potential. 48 sequences passed the minimum criteria and were categorized into three groups according to strength of evidence (sequences are color-coded in Additional File 1, Table S3, as indicated). Seven (7) are confirmed novel miRNAs since they had all the characteristics of known miRNAs and in addition also had a less abundant miR* sequence that maps on the opposite side of the stem from the putative novel miRNA. These are labelled green in Additional File 1, Table S3. Twenty-one (21) putative novel miRNAs are highly confident (labelled blue) since they also shared characteristics of known miRNAs but no sequence was found aligning to the miR* region. Given that the miR and miR* sequences for most known miRNAs have vastly different copy numbers such that the miR* sequence is sometimes not found, the highly confident candidates are also highly likely to be genuine novel miRNAs, Twenty (20) candidates (labelled grey) had a subset of the characteristics of known miRNAs but not all and therefore were deemed potential candidates that require more evidence.

Reads that did not align to known RNAs were assessed for miRNA potential through a novel miRNA discovery pipeline described by Creighton et al.[28] which tests for properties that are characteristic of known miRNAs. These properties include the following: 1) The mature sequence must map to the stem region of the hairpin sequence of the putative precursor extracted from the zebra finch genome. 2) The mature miRNA sequence must map to the precursor such that it can be processed following the Drosha processing rules [29]. All novel miRNA candidates that map to the loop region and/or lack appropriate Drosha processing sites are failed. 3) Known miRNAs have stable 5'-ends that vary at the most by +/- 1 nucleotide. 4) By contrast the 3'-ends of miRNAs are highly heterogeneous in length due to imprecise Dicer processing [29,30] and exhibit non-templated nucleotide sequence changes due to RNA editing [29-31]. 5) Consequently, the putative precursor must give a strong signal of sequence alignments in a tight area of 18-25 nucleotides. Small RNA sequences that are distributed fairly evenly along the entire length of the precursor are rejected since they likely represent degraded products of a large RNA. The candidates that also demonstrate the presence of the miRNA star sequence (miR*) mapping on the opposite side of the mature miRNA and occurring at a lower abundance in the deep sequencing data are considered to be confirmed novel miRNAs in zebra finch. Using this pipeline (Figure 1) we discovered 48 putative novel miRNAs that map on the zebra finch genome to a stem loop structure that folds with a minimum free energy of < -20 kcal/mol [32]. The complete analysis and mapping information for all the novel miRNA candidates is given in Additional File 1, Tables S2 and S3.

All novel miRNA candidates were mapped to genomic loci in the zebra finch genome assembly [33], and also to human and chicken genomes using the BLAT function of the UCSC Genome Browser (Additional File 1, Table S3). In the zebra finch genome, the loci include both annotated exons and introns as well as unannotated intergenic regions. Thirty-four (34) novel microRNAs uncovered from zebra finch are not presently detected in the human or chicken genome assemblies. Eleven (11) map to genome positions in chicken, and six to positions in the human (with three of these found in human but not chicken assemblies). Tgu-mir-2976 maps to three loci in the finch and 14 in the chicken, indicating a probable expansion of this miRNA in the chicken lineage. This putative novel miRNA is not currently detected in the human assembly HG18. Tgu-mir-2985 is intriguing as it is located within two stem loops within the introns of two functionally related genes: the glutamate receptor subunits GRIA2 and GRIA4 in all three genomes.

### miRNA responses to song exposure

When zebra finches are exposed to playback of a song they have not heard recently, changes occur in the expression of many different mRNAs as detected 30 min after stimulus onset [14]. To determine whether specific miRNAs also change in expression, we counted the Illumina reads in samples of RNA pooled from the auditory forebrain of birds either 30 min after onset of song playback (Song group) or from matched controls (Silence group). In our first such experiment, the birds in both groups were all males (n = 6 each). The read count for each miRNA in each sample was normalized to the total number of usable reads mapped in that sample. We then calculated the ratio of the normalized count in the Song-stimulated condition compared to the Silence condition and performed a Fisher's exact test (with correction for multiple testing) to evaluate whether the ratio differed significantly from the range of expected values at a 95% confidence interval. In the initial experiment with males, 49 of the known conserved miRNAs showed a significant difference, with 28 decreasing and 21 increasing in the group exposed to song (Additional File 1, Table S4).

To address the biological reproducibility of the miRNA responses to song more broadly, we then repeated the small RNA-seq comparison two additional times using new groups of birds. In the second experiment, we used only females, and in the third we used an equal mix of males and females. In total, therefore, we performed three independent "song-silence" pairwise comparisons by small RNA-seq, with an overall sex balance but different sex ratios in each individual comparison. These second and third experiments were done six months after the first and Illumina technology had improved by this time so that we obtained twice as many read counts (Table 1) - but again we normalized to the total mapped read number in each individual sample for our statistic analyses. As in the first experiment, we again observed differential read counts for roughly a third of the miRNAs, but the identities of the miRNAs affected were somewhat different in each comparison. This is summarized graphically as a Venn diagram (Additional File 2, Figure S1), and comprehensive read count data are presented in Additional File 1, Table S4. Across all three experiments, five conserved miRNAs showed changes that were both significant and in same direction in all comparisons (Table 2). For a number of other miRNAs, including let-7f, an apparent effect of song exposure was measured in all three experiments but the direction of change was not consistent (Additional File 1, Table S4).

**Table 2:** Conserved miRNAs with consistent responses to song exposure

	Male				Female				Mix			
	
	Silence	Song	Fold Change	FDR-P	Silence	Song	Fold Change	FDR-P	Silence	Song	Fold Change	FDR-P
	
*Increasing*												
tgu-miR-25	227	423	3.57	1.6E-27	55	212	3.60	1.4E-10	35	160	2.92	2.1E-05

tgu-miR-192	26	69	5.08	1.2E-06	36	90	2.33	5.5E-03	11	97	5.63	4.3E-06

*Decreasing*												

tgu-miR-92	359	100	0.53	1.1E-04	5479	5398	0.92	5.5E-03	7461	6887	0.59	6.3E-108

tgu-miR-124	24624	7056	0.55	2.1E-251	56802	46434	0.76	1.1E-206	50955	77220	0.97	1.6E-04

tgu-miR-129-5p	2020	602	0.57	4.0E-19	9778	7272	0.69	2.8E-62	12128	9284	0.49	2.6E-293

Shown are the Illumina read data for the five miRNAs that show a consistent response to song (same direction of change, significant in all three comparisons). "Song" and "Silence" list raw counts from the Illumina read analysis (Additional File 1, Table S4). "Fold Change" is the ratio of Song versus Silence read counts, after the raw counts were normalized within each run to the sum of mapped reads for that sample. Thus a value of > 1 indicates a relative increase in the group exposed to song, and < 1 indicates a decrease. "FDR-P" indicates the result of the Fisher's exact test (FDR adjusted) for this comparison. See Additional File 1, Table S4 for full list of values for all miRNAs, and associated TaqMan values for a subset of these miRNAs (measured in a different set of males and females).

We performed TaqMan assays on RNA from additional birds, probing for eleven of the "significantly affected" miRNAs, and obtained fluorescent signals in PCR for ten. In nine out of ten cases, we observed the same direction of song response by TaqMan as in the small RNA-seq experiment, although the P-value by TaqMan was below 0.05 in only five cases (tgu-miR-124, tgu-miR-29a, tgu-miR-92, tgu-129-5p, and tgu-miR-2954-3p, Additional File 1, Table S4). The lack of statistical significance in the TaqMan assay for the others could reflect differences in the sensitivity and resolution of Illumina vs. TaqMan assays, or the operation of other uncontrolled factors in our experiments that lead to variability in the expression of some miRNAs.

The transcriptional response in the auditory forebrain of *zenk *and other mRNAs is specific to song relative to non-song auditory stimuli [6,7,34,35]. To test for song-specificity of the miRNA response, we conducted a further TaqMan experiment assessing the levels of six miRNAs (tgu-miR-124, tgu-miR-92, tgu-miR-129-5p, and three miRNAs derived from the tgu-miR-2954 locus, next section), in birds who had heard either a normal song or a carefully matched non-song acoustic stimulus, "song enveloped noise" (SEN). SEN has the same amplitude envelope as the song from which it is derived but spectral content has been randomized so it does not sound like a song [34]. By TaqMan PCR, we confirmed that normal song induced a larger increase in *zenk *mRNA in these birds than did SEN (Additional File 2, Figure S3 panel D). In these same animals, normal song, but not SEN, triggered a significant decrease in the levels of tgu-miR-124, tgu-mir-129-5p, tgu-miR-92 and tgu-miR-2954-3p (Additional File 2, Figure S3 panels A-C, H). Thus we conclude that there is indeed a unique miRNA response in the auditory forebrain that is selective for song over non-song acoustic stimuli.

### A complex sex-linked miRNA locus in zebra finch and other birds

**Figure 2:**
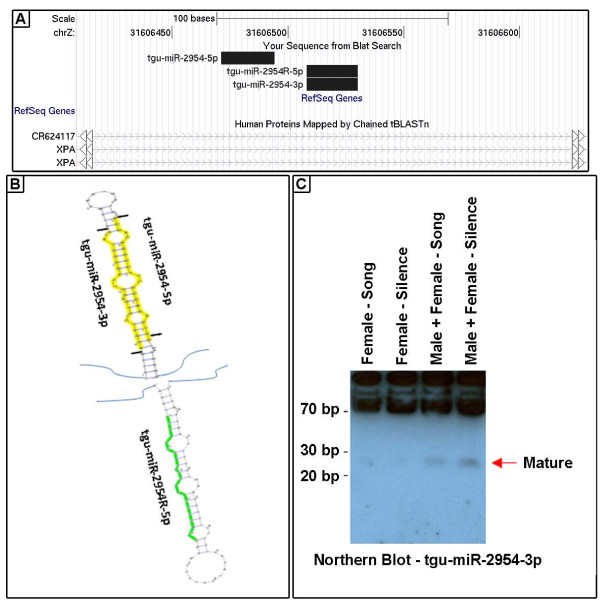
**The genome locus for tgu-mir-2954 produces three different miRNAs**. A. Alignments via the UCSC Genome Browser of the three detected miRNAs to the intron of the zebra finch XPA gene. B. Hairpin precursors for the three miRNAs. C. Northern blot analysis using an RNA probe complementary to novel miRNA tgu-miR-2954-3p.

The novel miRNA, tgu-mir-2954, that was detected most frequently in our Illumina assays maps to the sense strand of an intron in the XPA gene, on the Z chromosome (Figure 2A). The precursor hairpin contains reads from both arms, thus meeting our bioinformatic criteria for a confirmed miRNA (Figure 2B). By contrast to most known miRNAs, the numbers of reads from both 5' and 3' arms were found at similar copy numbers, suggesting that both arms may make functional mature miRNAs. BLAST analysis of the mir-2954 hairpin precursor sequence against the NCBI nr database identified a putative mature miRNA in chicken (gi|145279910|emb|AM691163.1|), and BLAT analysis of a collection of transcripts from crocodile and 11 other bird species [36] detected mir-2954 transcripts in 2 non-passerine species (two hummingbirds) and 3 passerine species (the American crow, the pied flycatcher, and the golden collared manakin) (Additional File 2, Figure S2). There was no BLAT hit in the crocodile, the remaining 3 non-passerine birds (Emu, budgerigar, and ringneck dove), and 3 passerine species (collared flycatcher, blue tit and Eastern phoebe). The lack of a hit does not necessarily mean absence of the gene as these datasets represent incomplete transcriptomes derived by 454 sequencing [36]. These results clarify that the sequence is not unique to the zebra finch or passerines, but may nevertheless have a restricted distribution within birds.

To validate the existence of these two miRNAs in zebra finch, we performed TaqMan analyses for both, using their reverse complements as controls. Interestingly, we got significant expression values not only for the predicted miRNAs but also for one of the reverse-complement miRNAs (tgu-miR-2954R-5p) although no significant song regulation for miR-2954R-5p was found (Additional File 2, Figure S3 panels I-J). With respect to the XPA gene within which this locus is embedded (Figure 2A), these data suggest that precursor-miRNA-stem loops are produced from both the sense (same orientation as XPA) and antisense strands. The stem loop precursor processed by Drosha from the sense RNA (tgu-mir-2954) generates two active miRNAs from its both arms (tgu-miR-2954-3p and tgu-miR-2954-5p). The stem loop precursor processed by Drosha from the antisense RNA (tgu-mir-2954R) generates at least one active miRNA (tgu-miR-2954R-5p) from its 5' end sequence.

We carried out Northern analysis on tgu-miR-2954-3p, which is the miRNA that has the highest number of read counts detected in our Illumina assays among the three miRNAs from the tgu-mir-2954 locus. A robust signal at ~22 nucleotides is evident in mixed-sex pools of RNA from birds hearing either song or silence, and a weaker signal is also detectable in two female-only pools of RNA (Figure 2C). Greater expression in males is consistent with the ZZ genotype of males and the lack of efficient sex chromosome dosage compensation in the zebra finch [37,38].

**Figure 3:**
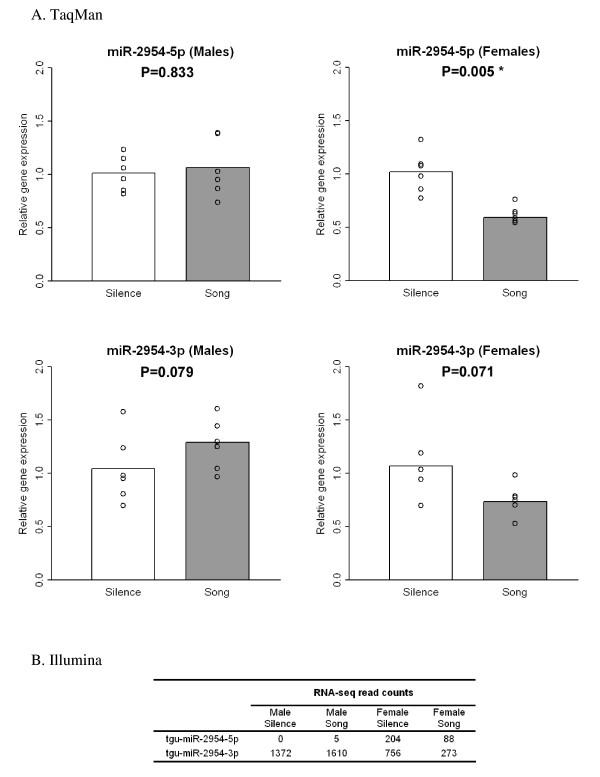
**Analysis of miRNAs produced at the tgu-mir-2954 locus**. TaqMan and Illumina RNA-seq data generated from independent sets of birds (n = 6 in each data set) for expression from the tgu-mir-2954 locus. A) TaqMan results, where the relative gene expression of each individual bird (open circle) was obtained by using the 2^-ddCt method [98]; the relative gene expression of either Silence (white bar) or Song (gray bar) group was the mean of six individuals; the P value was calculated by paired t test since each song stimulated animal was explicitly paired with a silence control animal collected simultaneously. B) Read counts from the Illumina RNA-seq for miR-2954-3p and miR-2954-5p (also shown in the Additional File 1, Table S4).

By TaqMan as well as by Illumina, we observed an apparent sex difference in the direction of the response of tgu-miR-2954-3p to song - up in males and down in females (Figure 3 and Additional File 1, Table S4). This suggests this locus may be under complex regulation, integrating information about sex, auditory or social experience and perhaps also other factors related to XPA gene expression.

To gain insight into the potential functional role of tgu-miR-2954-3p in the response to song, we used a conservative strategy to predict gene targets that are both conserved in birds and responsive to song exposure in the zebra finch. Potential targets of miRNAs are described as mRNAs that have sequences that can undergo Watson-Crick base pairing with the 5'-seed (nucleotide 2-7) of the miRNA [39]. For target prediction we applied the TargetScan (5.1) algorithm using the chicken genome as an initial reference, and then confirmed presence of the target sequence in the zebra finch. For evidence of song responsiveness, we used the data set of Dong et al. [14]. Eight genes met all these criteria (Table 3) and are thus both song-responsive and also subject to regulation by tgu-miR-2954-3p. These genes all have functions in control of cell proliferation or neurite outgrowth (see below).

**Table 3:** Song-regulated targets of tgu-miR-2954-3p

Ensembl ID	Gene Symbol	EST	Gene Name
ENSTGUG00000001349	ELAVL2	CK313262	ELAV-like protein 2 (Hu-antigen B)(HuB)(ELAV-like neuronal protein 1)(Nervous system-specific RNA-binding protein Hel-N1)

ENSTGUG00000001404	LINGO2	DV957508	Leucine-rich repeat and immunoglobulin-like domain-containing nogo receptor-interacting protein 2 Precursor (Leucine-rich repeat neuronal protein 6C)(Leucine-rich repeat neuronal protein 3)

ENSTGUG00000003073	TLK2	CK305975	Serine/threonine-protein kinase tousled-like 2 (EC 2.7.11.1)(Tousled-like kinase 2)(PKU-alpha)

ENSTGUG00000008207	BTG1	CK303273	Protein BTG1 (B-cell translocation gene 1 protein)

ENSTGUG00000008540	CHD2	DV958991	Chromodomain-helicase-DNA-binding protein 2 (CHD-2)(EC 3.6.1.)(ATP-dependent helicase CHD2)

ENSTGUG00000010181	XP_002196848.1	CK304764	crk-like protein (v-crk avian sarcoma virus CT10 oncogene homolog-like) (CRKL)

ENSTGUG00000010364	NEGR1	DV954047	Neuronal growth regulator 1 Precursor

ENSTGUG00000011700	HMGB1	CK314519	High mobility group protein B1 (High mobility group protein 1)(HMG-1)

We used TargetScan to find binding sites of tgu-miR-2954-3p on eight chicken genes and here are listed the information of their homologous genes in the zebra finch genome including Ensembl IDs, Gene Symbols, EST (Accession numbers of song-regulated EST identified in the previous microarray study) and Gene Names (or aliases in parenthesis).

## Discussion

Here we show that a natural perceptual experience, hearing the sound of another bird singing, alters the profile of miRNAs in parts of the songbird brain responsible for auditory perception, integration and memory. The song-regulated population includes both known (conserved) and novel miRNAs. We highlight one sex-linked song-responsive miRNA and identify mRNAs that are potential targets of its action during song exposure. Thus miRNAs may have roles in the information processing functions of the brain, in addition to their roles in brain development and evolution.

To demonstrate this, we first catalogued the miRNAs expressed in the adult zebra finch auditory forebrain. We used massively parallel Illumina sequencing of small RNAs to perform this cataloguing efficiently. In addition to known conserved miRNAs, our analysis identified 48 small RNA sequences that meet the structural criteria for miRNAs but had not been described in miRBase in any organism at the time of our analysis. Fourteen of these are detected in the chicken or human genome assemblies and may give rise to miRNAs that have not yet been described elsewhere due to low copy number, restricted tissue distribution or other factors. The remaining novel miRNAs, 34 in number, may be unique to the zebra finch or the songbird lineage. Few studies have attempted *de novo *identification of miRNAs from the brain [18] and ours is the first to report direct sequencing of songbird brain miRNAs. A previous study did identify precursor sequences for five conserved miRNAs in the developing zebra finch brain [40]. Also, in parallel with our own Illumina analysis, Li and her colleagues used 454 sequencing to identify miRNAs in the brain and liver of adult zebra finches. These different sets of annotations are compared and collated in a supplement to the analysis of the zebra finch genome assembly [33].

By comparing birds hearing novel song playbacks or silence, we found evidence for experience-dependent fluctuations in large numbers of miRNAs in the auditory forebrain. We performed three separate pairwise comparisons by Illumina, where all aspects of the experimental conditions were carefully counterbalanced between the two groups in each comparison. The three comparisons were not direct replications of each other, as each had a different sex ratio. Our reasons for varying the sex ratio were partly pragmatic (limited numbers of birds of the same sex that could be removed from our aviary) and partly analytical (males and females have different behavioral responses to songs). Some of the differences between the three sets of results may reflect real biological differences in the responses of males and females. Indeed, our Northern analysis of the tgu-miR-2954-3p confirms a sex difference in expression of this Z-linked miRNA gene. This is especially intriguing because we also obtained TaqMan evidence for both sense and antisense transcripts of this miRNA. One can imagine scenarios where different ratios of sense and antisense transcription occur in males (two copies of the gene) and females (one copy of the gene) with different consequences on the transcriptional networks affected by song exposure in the two sexes.

Ignoring the potential effects of sex, we identified five miRNAs that showed significant and consistent changes in response to song across all three Illumina comparisons. Three miRNAs consistently decreased after song (tgu-miR-92, tgu-miR-124, tgu-miR-129-5p) and two increased (tgu-miR-25, tgu-miR-192). The down-regulated miRNAs are at much higher abundance (> 1000 reads in each run) and perhaps for this reason we were more successful at detecting them and replicating their song regulation by TaqMan assay in subsequent experiments with additional groups of birds. The most abundant miRNA in our regulated set, tgu-miR-124, consistently met the statistical test for significant down-regulation by song, in each of six separate experiments (three Illumina comparisons, two TaqMan analyses in Additional File 1, Table S4, and the TaqMan comparison of song vs. SEN in Additional File 2, Figure 3).

In studies in other species, miR-124 has been linked to brain plasticity and development in several contexts. Chronic cocaine administration results in down-regulation of miR-124 in the rodent mesolimbic dopaminergic system [41]. In the developing chick neural tube, miR-124a is a component of a regulatory network that controls the transition between neural progenitors and post-mitotic neurons [42]. miR-124 also regulates adult neurogenesis, and its overexpression promotes neuronal differentiation [42,43] and neurite outgrowth [44]. Intriguingly, in songbirds neurogenesis continues in the forebrain throughout adulthood, from a population of precursor cells that line the walls of the lateral ventricles and have the characteristics of neural stem cells [45-47]. The net rate of neuronal addition and loss in the adult songbird has been shown to depend on social and environmental influences [48-51]. Perhaps tgu-miR-124 is a regulatory link between experience and neurogenesis - further study of this fascinating possibility is clearly warranted.

Although miRNAs can have diverse functions, they often act by altering the concentrations of specific mRNAs they target via complementary base pairing. We used the TargetScan algorithm [52] to predict binding sites of tgu-miR-2954-3p in chicken genes, and then we confirmed the presence of the same conserved target sequence in the zebra finch genome assembly. We found eight targets that met these criteria and were also regulated by song in the Dong et al. microarray data [14]. These eight genes have a provocative coherence in their function, as they are all implicated in control of cell proliferation and neuronal differentiation. Six operate by affecting gene expression and chromatin remodeling as we briefly review here. ELAVL2 is a member of a protein family that binds AU-rich regions in the 3'UTR of genes such as c-fos and promotes the shift from cell proliferation into cellular differentiation [53-57]. TLK2 is a kinase tightly associated with DNA replication during cell division [58]. At least one of its targets, the histone chaperone Asf1, controls chromatin assembly, thus TLK2 activity can regulate transcription and elongation [59-61]. BTG1 is also regulated during the cell cycle [62]. It acts as a cofactor for Hoxb9, a transcription factor that controls cell proliferation and differentiation, and BTG1 reduces rates of cell proliferation [62-64]. CHD2 can potentially affect transcription of many genes by remodeling chromatin [65,66]; disruption of CHD2 has profound consequences for development and is implicated in many human diseases [67-69]. HMGB1 is another DNA binding protein that facilitates transcription by altering chromatin structure to ease promoter binding [70-73]. Some of the genes regulated by HMGB1 may play a role in cell proliferation and migration [74,75]. Neuronal migration and neurite outgrowth are affected by CRKL, a transcriptional activator that is a component of the reelin pathway [76-79]. Unlike the other six genes, NEGR1 and LINGO2 do not seem to alter transcription but they do have established roles in neuronal differentiation. NEGR1 affects cell-cell adhesion to modulate neurite outgrowth and synapse formation [80-82]. LINGO2 is one member of a family of transmembrane proteins that are involved in neural and axonal regeneration [83,84]. The function of LINGO2 is untested, but expression of a related protein, LINGO1, is attenuated in cortical areas deprived of sensory input and is a partner in a signaling pathway that correlates with neuronal activity during a learning paradigm [85,86].

## Conclusions

In conclusion, these data reveal a network of miRNAs in the zebra finch's auditory forebrain, responsive to the experience of hearing another bird sing. The network includes well-characterized conserved miRNA known to have roles in neuronal differentiation (miR-124), and novel miRNAs that can target genes that control neuronal differentiation (tgu-miR-2954-3p). Our data suggest this miRNA network may influence the fundamental shift we have observed in the transcriptional and metabolic state of the auditory forebrain during the process of song-specific habituation [14,87]. Further study of song responses in the zebra finch may reveal general insights into the neurogenomic mechanisms that underlie learning, memory and the ongoing adaptation to experience.

## Methods

### Song stimulation and brain dissections

Zebra finches were obtained from aviaries maintained at the University of Illinois. All procedures involving animals were conducted with the approval of the University of Illinois Institutional Animal Care and Use Committee. The birds were raised in a standard breeding aviary and were tutored under normal social conditions (i.e., by their parents or other adult birds in the breeding colony). All birds used in this study were adults (older than 90 days after hatching). The song playback procedures and brain dissections were performed exactly as in previous microarray analyses, using the same equipment [14,88]. Briefly, each bird was put individually into a sound isolation chamber for 18 hours on the first day, and on the second day those in the song group heard 30 minutes of a song not heard previously ("novel song"). Matched controls collected in parallel heard no song playback ("silence"). Birds were sacrificed in song-silence pairs, so that 5 minutes before the end of the song playback to one bird, a bird in the silence group was sacrificed and its auditory forebrain was dissected and frozen in dry ice. Then the auditory forebrain of the song-stimulated bird was dissected and frozen in dry ice. The auditory forebrain dissection (also referred to as auditory lobule) is described in [89] and collects NCM (caudomedial nidopallium), CMM (caudomedial mesopallium) and the enclosed Field L subregions. At the end of the song stimulation procedure, all auditory forebrains were transferred and stored at -80C until RNA isolation. For the comparison of responses after overnight isolation to song versus SEN (Additional File 2, Figure S3), we used two matched stimuli derived from bird "C7" as previously described [34].

### RNA Samples

For Illumina analyses: Total RNA was extracted using the mirVana miRNA Isolation Kit (Ambion) from three pairs of pooled auditory forebrain samples. 1) Males (samples S7 and S8): 6 birds per pool, collected in November 2008. 2) Females (samples S1 and S2): 6 birds per pool, collected in May 2009. 3) Mixed (samples S3 and S4), 3 males and 3 females each pool, collected in May 2009. Samples with odd numbers were from birds hearing song, and even number hearing silence.

For Northern analysis: Auditory forebrains of 22 birds (12 females and 10 males) were collected in April 2009, and total RNA was extracted by Tri-Reagent (Ambion). Male and female samples were pooled after extraction.

For TaqMan analysis: Analyses were performed on total RNA extracted either by mirVana or Tri-Reagent (Ambion), from the auditory forebrains of individual males or females, collected in April-August 2009, March 2010 or December 2010.

### Illumina small RNA sequencing and novel miRNA discovery

Fifteen micrograms of total RNA from auditory forebrain of song bird samples described above were gel-fractionated to isolate 18-30 nt small RNAs. 3' and 5' adapters were ligated to the small RNAs and constructs amplified following RT-PCR following the conditions specified in the small RNA kit (FC-102-1009, Illumina) protocol. The small RNA library was sequenced using a Solexa/Illumina GA-1 Genome analyzer. Small RNA sequences were analyzed through a high-throughput computational pipeline described by [28,29,90,91]. To identify zebra finch miRNAs that are also conserved in chicken, human and mouse, we performed a local Smith-Waterman alignment of each unique sequence read against each of the mature miRNAs in miRBase version 13.0 for each of these species. We allowed for a 3 base overhang on the 5' end and a 6 base overhang on the 3' end. In the case of redundantly aligning reads, mature miRNA sequences were equally apportioned among each of the hairpins. For each sample, all sequence reads were aligned to a reference set of precursor miRNAs from miRBase version 13.0. The reads that did not align to any known miRNA were passed to our novel miRNA discovery platform as previously described [28]. Briefly, each sequence is first mapped to the reference genome sequence (WUGSC 3.2.4) and 200 bases of flanking sequence are extracted to further define the putative hairpin. This extracted sequence is then folded using the Vienna RNA folding package [92] and those sequences that form a plausible hairpin are selected as potential novel miRNA hairpins. These candidates are filtered through a set of three Ambros criteria: 1) the mature putative miRNA sequence must rest on one side of a single hairpin; 2) the putative miRNA sequence must bind relatively tightly within the hairpin stem containing no large or energetically unfavorable loops; and 3) the putative hairpin must have a miRNA-appropriate energy (free energy below -20 kcal/mol). All sequences that passed were then carefully curated to determine if Drosha and Dicer processing could yield the resulting mature sequence from the predicted hairpin. These candidates are then divided into four different categories: "not likely", "potential", "high confidence", and "confirmed" (as in red, gray, blue and green colors in Additional File 1, Tables S2 and S3). Candidates that are flagged red as "not likely" either failed to map in a pile of sequences in a very tight space of 15-25 nt of the predicted hairpin (e.g. were scattered evenly across the full length of the hairpin), mapped within the loop of the hairpin, or mapped to known tRNAs or rRNAs. Candidates that passed all of the above criteria, and also mapped within a hairpin with predicted Drosha and Dicer cut sites were categorized as "high confidence" (blue annotation in Additional File 1, Tables S2 and S3). All high confidence candidates for which we detected both the mature sequence and the putative star sequence from the same hairpin we categorized as "confirmed" (green annotation in Additional File 1, Table S3). In addition to miRNA precursors, the reads were also mapped to the reference zebra finch genome using the Pash software package [93,94], and uploaded to the Genboree platform (http://www.genboree.com) to identify potential mappings to piRNAs, snoRNAs and other annotations in addition to miRNAs (data shown in Additional File 1, Table S1). PiRNAs (i.e., Piwi-interacting RNAs) have a central role in the maintenance of the integrity of genomes through the silencing of transposable elements [95]. SnoRNAs (small nucleolar RNAs) function in site-specific ribosomal RNA modification, rRNA processing and more recently have been found to guide alternate splicing and RNA editing of mRNA transcripts [96].

### TaqMan qPCR

To measure the mature miRNA, the TaqMan MicroRNA Assay Kit (Applied Biosystems) was used according to the manufacturer's instructions. Probe sequences used for each target miRNA are given in Table 4.

**Table 4:** Probes used for Taqman analysis of specific miRNA sequences

miRBase name	Company name	Sequence detected
tgu-let-7a	let-7a	5'-UGAGGUAGUAGGUUGUAUAGUU-3'
tgu-let-7f	let-7f	5'-UGAGGUAGUAGAUUGUAUAGUU-3'
tgu-miR-124	miR-124	5'-UAAGGCACGCGGUGAAUGCC-3'
tgu-miR-9	miR-9	5'-UCUUUGGUUAUCUAGCUGUAUGA-3'
tgu-miR-129-5p	miR-129-5p	5'-CUUUUUGCGGUCUGGGCUUGC-3'
tgu-miR-129-3p	miR-129-3p	5'-AAGCCCUUACCCCAAAAAGCAU-3'
tgu-miR-29a	miR-29c	5'-UAGCACCAUUUGAAAUCGGU-3'
tgu-miR-92	miR-92a	5'-UAUUGCACUUGUCCCGGCCUGU-3'
tgu-miR-25	miR-25	5'-CAUUGCACUUGUCUCGGUCUGA-3'
RNU6B	RNU6B	5'-CGCAAGGAUGACACGCAAAUUCGUGAAGCGUUCCAUAUUUUU-3'
tgu-miR-2954-5p	novel51F-5p	5'-GCUGAGAGGGCUUGGGGAGAGGA-3'
tgu-miR-2954-3p	novel51F-3p	5'-CAUCCCCAUUCCACUCCUAGCA-3' (Northern validated)
tgu-miR-2954R-5p	novel51R-5p	5'-UGCUAGGAGUGGAAUGGGGAUG-3'
tgu-miR-2954R-3p	novel51R-3p	5'-UCCUCUCCCCAAGCCCUCUCAGC-3'

### Northern Blot Analysis

Northern blotting to confirm novel miRNA tgu-miR-2954-3p was performed by modifying the protocol of [97]. 2 μg of total RNA was heated at 65°C for 5 min with 2X loading dye (Ambion), quenched on ice, and loaded on a 15% TBE Urea gel (Invitrogen). Total RNA was separated by electrophoresis at 200V for 50 min. The gel was stained with with EtBr in 1x TBE (4 μL of 10 mg/ml EtBr per 100 ml of 1x TBE) for 3 minutes with gentle shaking and transferred to nylon membrane for 90 min at 200V using 1X TBE buffer at room temperature. The membrane was cross-linked at 1200 kJ for 45 seconds. RNA probes were synthesized for tgu-miR-2954-3p probe 5' - UGCUAGGAGUGGAAUGGGGAU G - 3' by Integrated DNA Technologies. Radio labeling was carried out in a reaction of 12.0ul dH2O + 2.0ul PNK buffer + 1.0ul (100ng/ul) probe + 1.0ul PNK polymerase (Promega) + 4.0ul P^32^-gamma-ATP (10mCi/ml) (PerkinElmer). The reaction was incubated at 37°C for 1 hour and inactivated at 65°C for 10 min. The probe was purified using Nick columns from GE following manufacturer's instructions. The membranes were pre-hybridized for 30 min with 20 ml of pre-hybridization buffer (5X SSC + 20 mM NaPO4 + 7X SDS + 2X Denhardt (pre warmed) at 60° C) in a rotating hybridization oven. Hybridization was carried out at 50°C in a rotating incubator for 24h. The membranes were washed for 10 min at 50°C with 20-30mL of wash buffer (2X SSC + 0.5% SDS). When background was ~0.5 cpm, the membranes were wrapped in saran wrap and exposed at -80°C for ~72h.

## Authors' contributions

PHG coordinated the work of Illumina RNA-seq and prepared the manuscript. YL conducted the song exposure experiments and subsequent dissections and RNA extractions, performed TaqMan qPCR, analyzed differentially expressed miRNAs and participated in drafting the manuscript. ALB performed Illumina RNA-seq and Northern blot. JD helped analyze expression data of Illumina RNA-seq and TaqMan qPCR. CC, JBT, CJC, JHK, and AM participated in mapping and analyzing Illumina RNA-seq data. MW and SGJ helped with miRNA sequence annotation. DFC designed and coordinated the study and drafted the manuscript. All authors read and approved the manuscript.

## Supplementary Material

Additional file 1 **Supplemental tables.xls**. This one file contains all four Supplemental Tables, each as a separate worksheet. **Table S1 ("1 overview") **is a summary of Illumina sequence read alignments for six pools of RNA from zebra finch auditory forebrain responding to song versus silence, and shows the distribution of sequence reads in relation to multiple genomes and multiple annotations in the current genomic databases. **Table S2 ("2 novel hairpins") **gives detailed alignments of putative pre-miRNAs and read sequences. **Table S3 ("3 novel genes") **shows annotations of all novel miRNA loci mapped in genome assemblies of zebra finch, chicken or human. **Table S4 ("4 all read counts") **gives read counts and current annotation in miRBase of all conserved and novel miRNAs, with statistics. Click here for file

Additional file 2 **Supplemental figures.doc**. This one file contains all three supplemental figures. **Figure S1 **is a Venn diagram of numbers of miRNAs with significant differential expression in response to novel song in three Illumina experiments. **Figure S2 **shows a comparative mapping in other avian transcriptomes of tgu-mir-2954. **Figure S3 **demonstrates the song-specificity of the miRNA response, using TaqMan to compare the levels of specific miRNAs in animals from groups that heard song, matching song-enveloped noise, or silence. Click here for file
