# Supplementary material for: Song exposure regulates known and novel microRNAs in the zebra finch auditory forebrain
Source: BMC Genomics. 2011 May 31;12:277. doi: 10.1186/1471-2164-12-277 (PMC3118218; doi:10.1186/1471-2164-12-277)
Supplement: Additional file 2 — Supplemental figures.doc. This one file contains all three supplemental figures. Figure S1 is a Venn diagram of numbers of miRNAs with significant differential expression in response to novel song in three Illumina experiments. Figure S2 shows a comparative mapping in other avian transcriptomes of tgu-mir-2954. Figure S3 demonstrates the song-specificity of the miRNA response, using TaqMan to compare the levels of specific miRNAs in animals from groups that heard song, matching song-enveloped noise, or silence. [file 1471-2164-12-277-S2.DOC]

**Figure S1. Venn diagram of numbers of miRNAs with significant differential expression in response to novel song in three Illumina experiments.**

1. Increased in Song compared to Silence


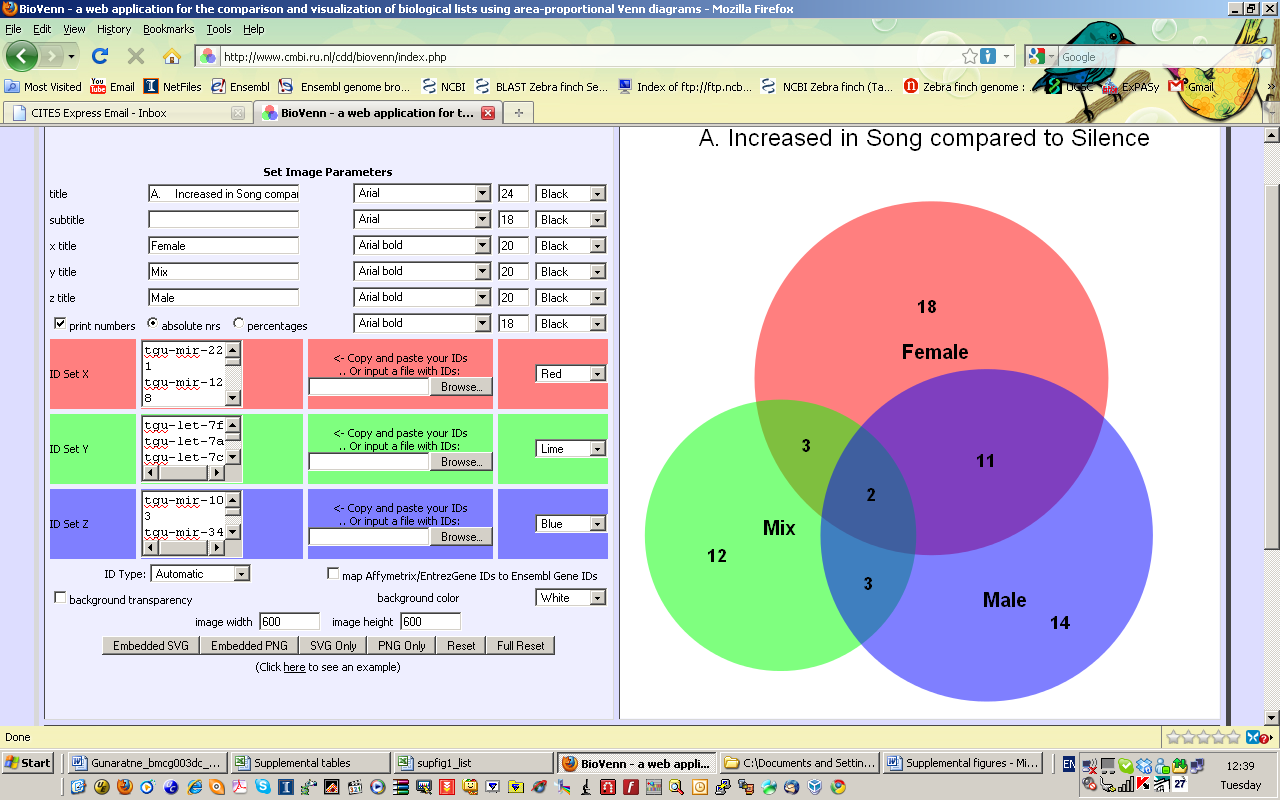


1. Decreased in Song compared to Silence


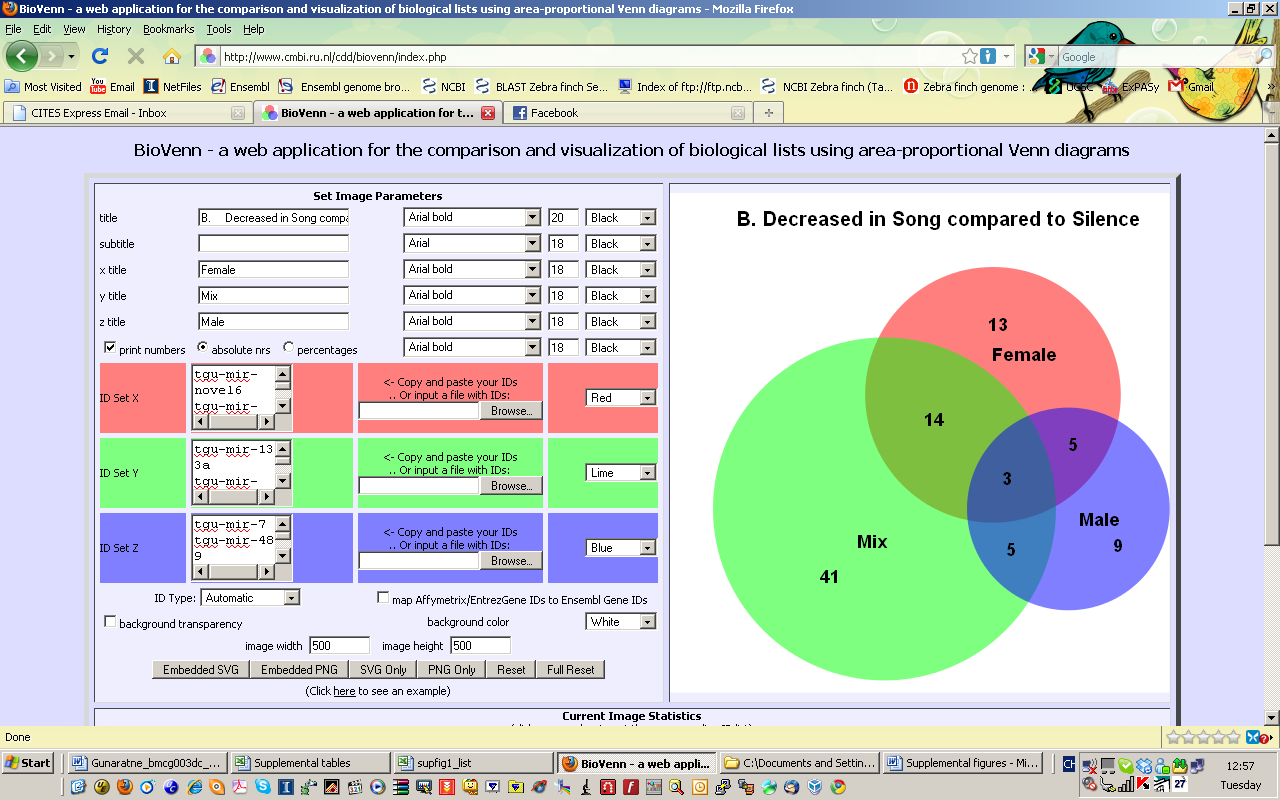


**Figure S2. Comparative mapping in other avian transcriptomes of tgu-mir-2954.** cDNA from other species (34) was sequenced (Roche 454) and alignments of tgu-2954 are shown at two levels of magnification: A) the nucleotide level for the mature miRNA sequence; B) the level of the larger XPA gene in which the miRNA is embedded.

**A. Nucleotide level alignment**


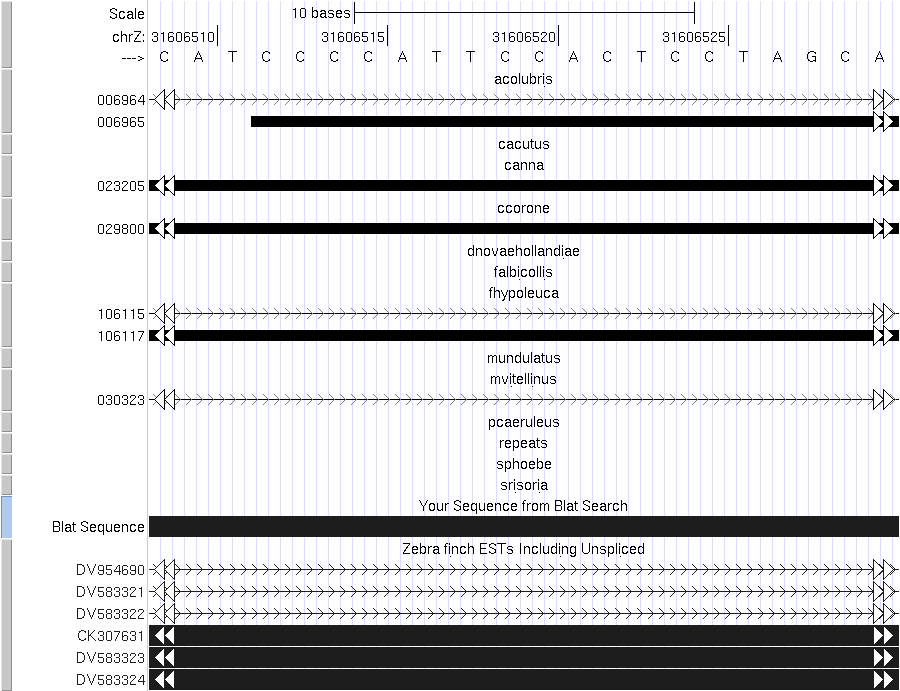


**B. XPA gene level alignment**


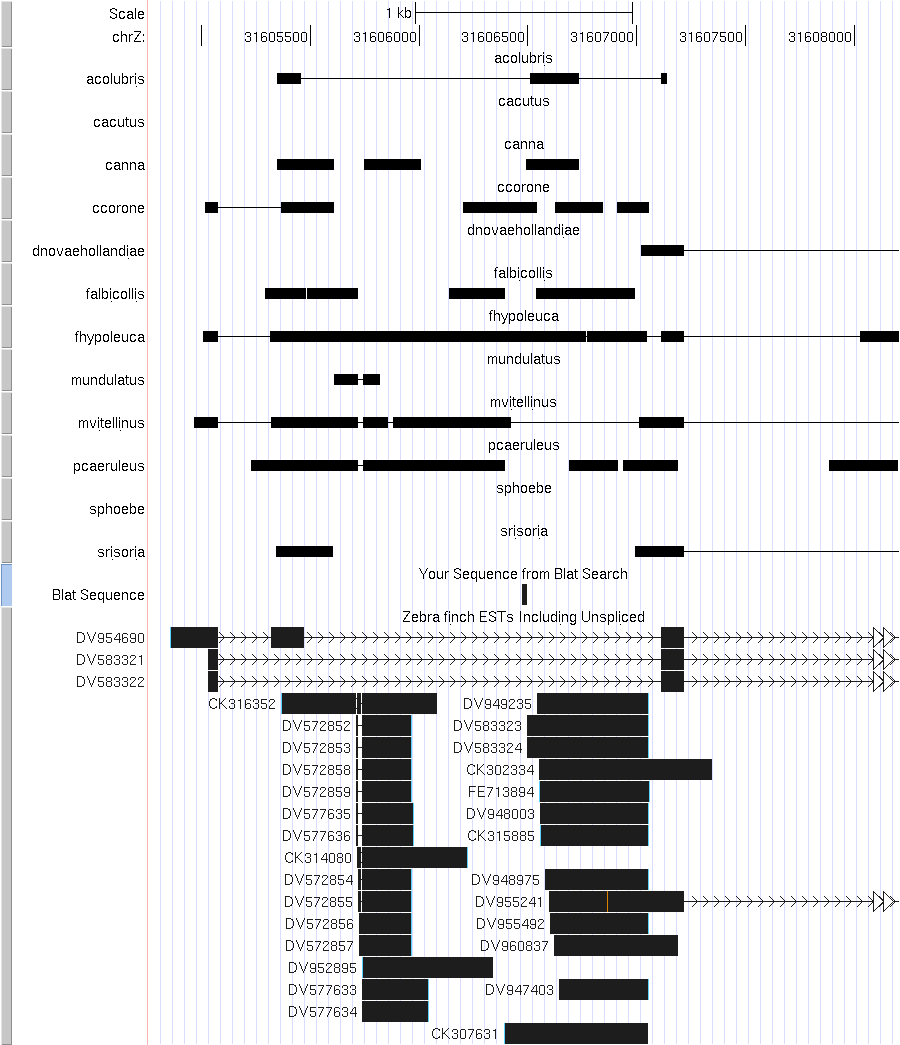


**Figure S3. Song-specificity of the miRNA response** (panels on the following 3 pages**).**

TaqMan was used to compare the levels of specific miRNAs in animals from three treatment groups. One group heard silence, another heard the normal song of bird C7 (C7song), and the third group heard a matched non-song stimulus derived by randomizing the spectral content of C7 (C7noise) . Relative gene expression in each individual bird was obtained by using the 2^-ddCt method and presented as a dot in the plot. The relative gene expression mean of each group of birds is shown by the bar. Results of statistical analyses (ANOVA followed by Tukey’s HSD posthoc tests) for each miRNA are listed below each plot.

**Figure S3, cont.**

| A | B |
| --- | --- |
| 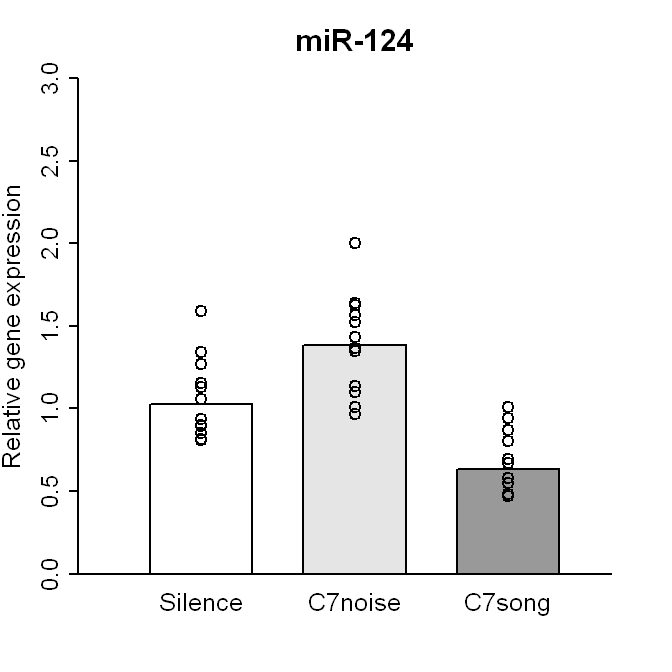 | 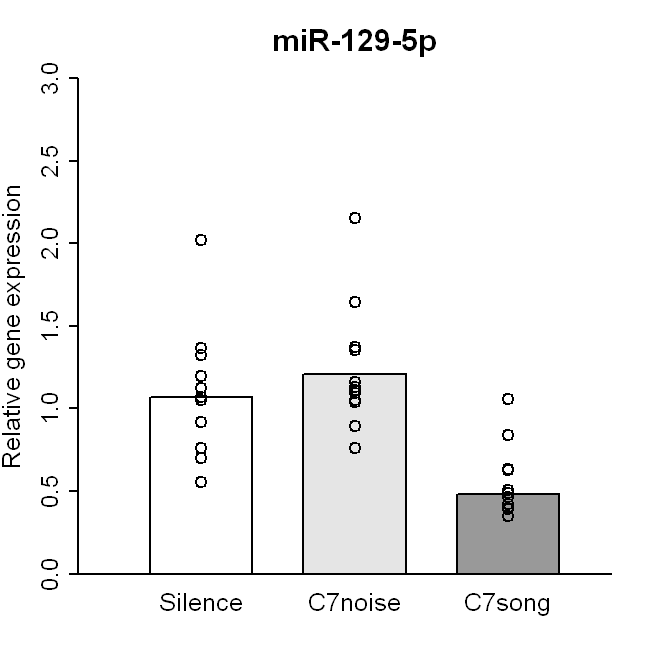 |
| **ANOVA** p = 1.237e-07 ***  **Post hoc analyses after ANOVA**  C7noise vs. Silence p = 0.0297968  C7song vs. Silence p = 0.0001966  C7song vs. C7noise p = 0.0000001 | **ANOVA** p = 4.046e-07 ***  **Post hoc analyses after ANOVA**  C7noise vs. Silence p = 0.5995880  C7song vs. Silence p = 0.0000154  C7song vs. C7noise p = 0.0000009 |
| C | D |
| 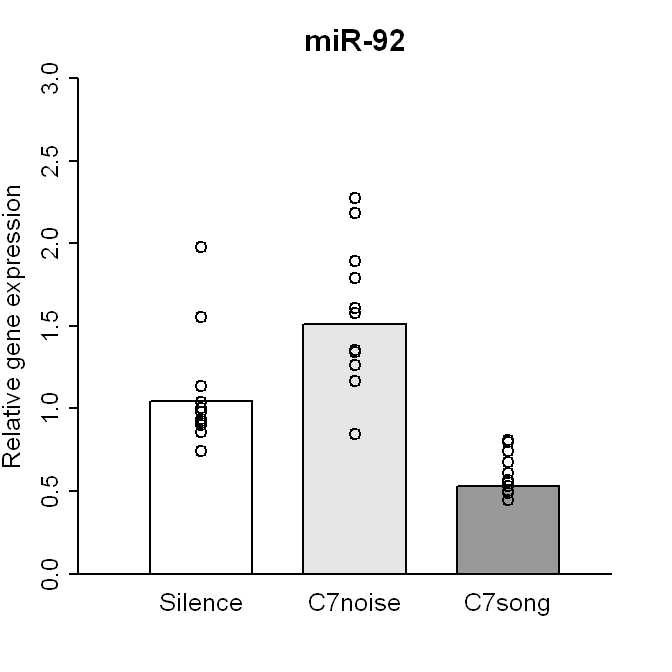 | 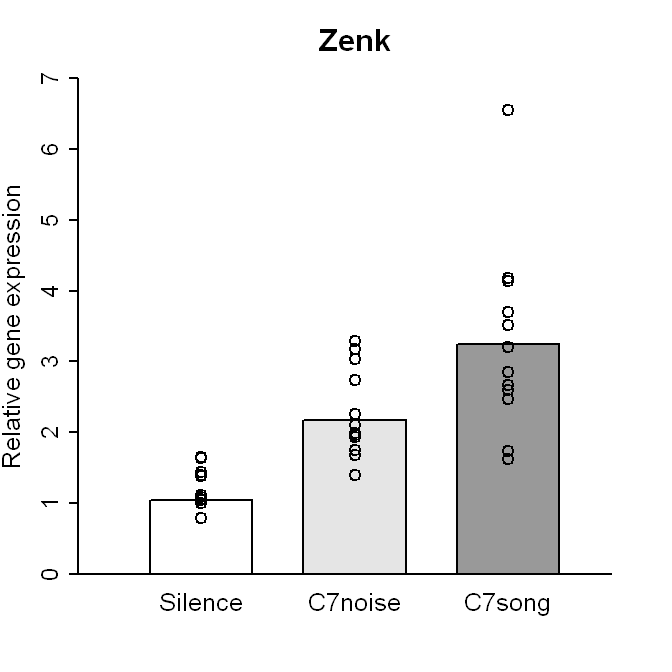 |
| **ANOVA** p = 6.287e-09 ***  **Post hoc analyses after ANOVA**  C7noise vs. Silence p = 0.0176403  C7song vs. Silence p = 0.0000175  C7song vs. C7noise p = 0.0000000 | **ANOVA** p = 2.516e-08 ***  **Post hoc analyses after ANOVA**  C7noise vs. Silence p = 0.0000317  C7song vs. Silence p = 0.0000000  C7song vs. C7noise p = 0.0386849 |

**Figure S3, cont.**

| E | F |
| --- | --- |
| 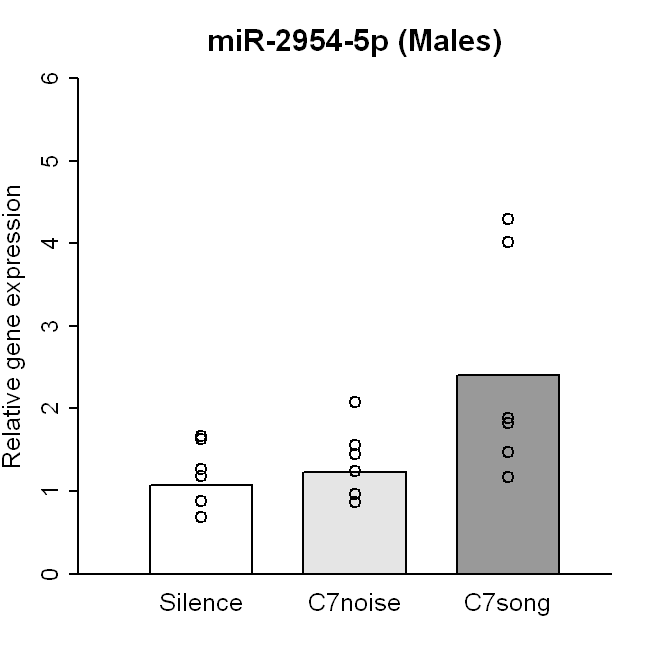 | 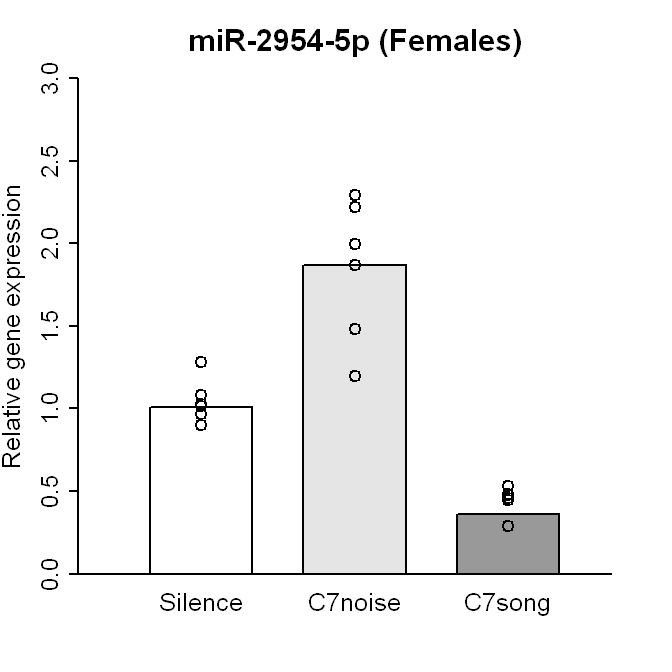 |
| **ANOVA** p = 0.04574 *  **Post hoc analyses after ANOVA**  C7noise vs. Silence p = 0.8724359  C7song vs. Silence p = 0.0494895  C7song vs. C7noise p = 0.1228024 | **ANOVA** p = 2.696e-08 ***  **Post hoc analyses after ANOVA**  C7noise vs. Silence p = 0.0020331  C7song vs. Silence p = 0.0000063  C7song vs. C7noise p = 0.0000000 |
| G | H |
| 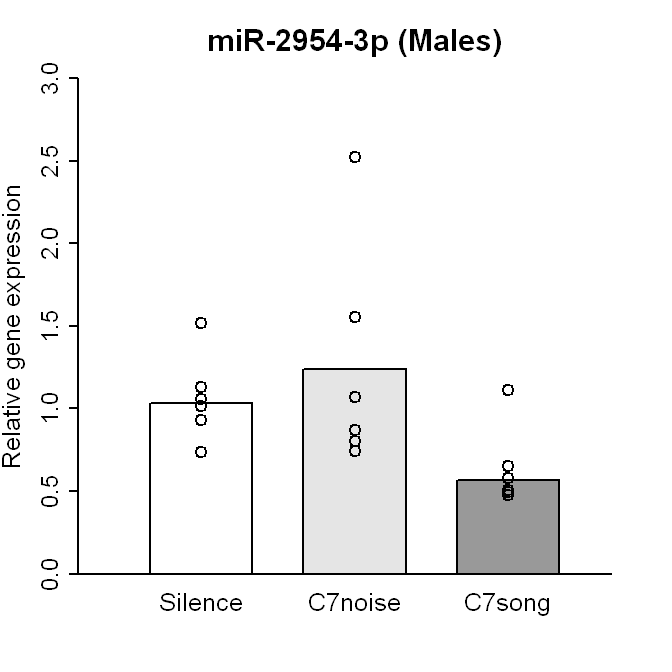 | 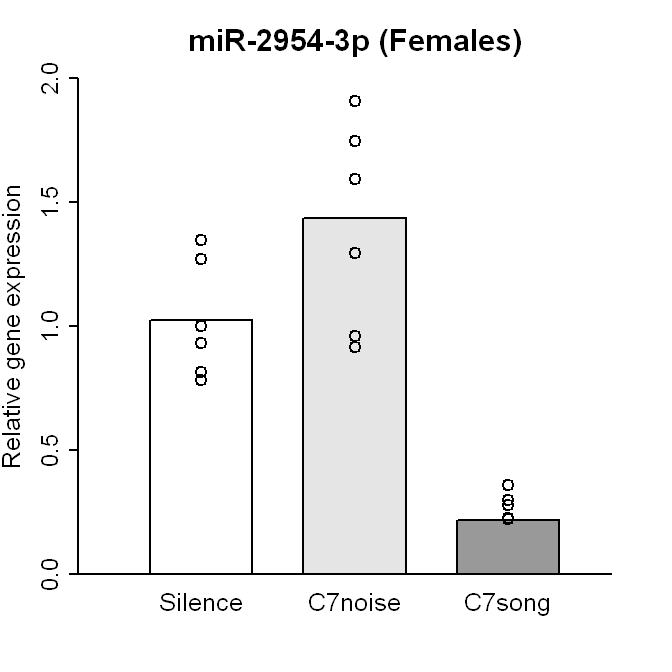 |
| **ANOVA** p = 0.01287 *  **Post hoc analyses after ANOVA**  C7noise vs. Silence p = 0.9140920  C7song vs. Silence p = 0.0372510  C7song vs. C7noise p = 0.0168752 | **ANOVA** p = 1.251e-08 ***  **Post hoc analyses after ANOVA**  C7noise vs. Silence p = 0.1521374  C7song vs. Silence p = 0.0000002  C7song vs. C7noise p = 0.0000000 |
| **Figure S3, cont.** |  |
| I | J |
| 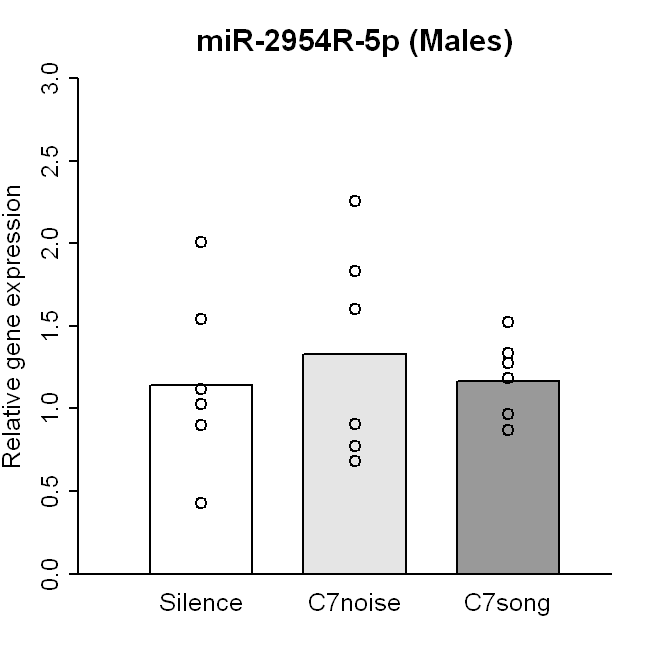 | 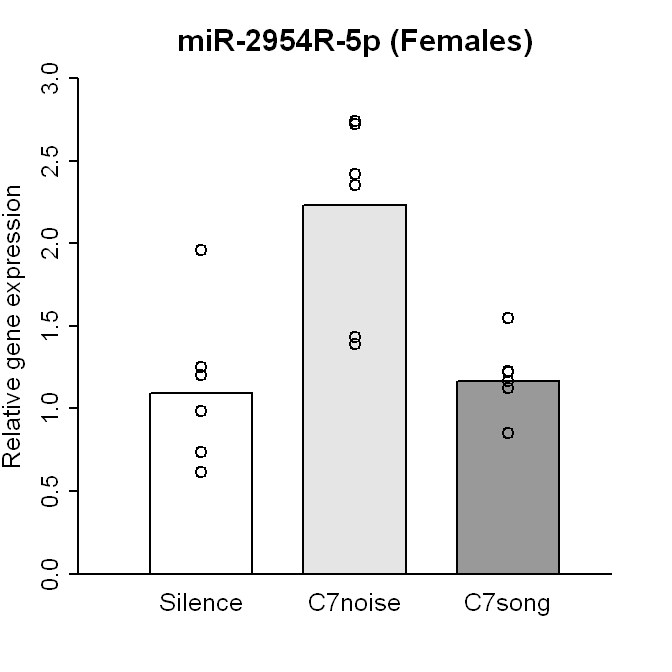 |
| **ANOVA** p = 0.8366 | **ANOVA** p = 0.004361 **  **Post hoc analyses after ANOVA**  C7noise vs. Silence p = 0.0052666  C7song vs. Silence p = 0.7939114  C7song vs. C7noise p = 0.019361 |
